# Inhibitory brainstem reflexes under external emotional-stimuli in bipolar I and II disorders

**DOI:** 10.1186/s12888-017-1390-3

**Published:** 2017-06-19

**Authors:** Qisha Zhu, Jiawei Wang, Chanchan Shen, Hongying Fan, Bingren Zhang, Guorong Ma, Yanxia Lu, Wei Wang

**Affiliations:** Department of Clinical Psychology and Psychiatry/ School of Public Health, Zhejiang University College of Medicine, Yuhangtang Road 866, Hangzhou, Zhejiang 310058 China

**Keywords:** Bipolar I and II disorders, Brainstem reflex, External emotional-stimuli, Temporalis exteroceptive suppression

## Abstract

**Background:**

Bipolar disorder types I (BD I) and II (BD II) might present different dysfunctions of the cortex and brainstem, as reflected by the second exteroceptive suppression period of temporalis muscle activity (ES2) under different stimuli of external emotions.

**Methods:**

This study included 30 BD I and 20 BD II patients, and 40 healthy volunteers. All participants were invited to answer the Mood Disorder Questionnaire, the Hypomania Checklist-32, and the Plutchik-van Praag Depression inventory, as well as to undergo the ES2 test under external emotional-stimuli (emotional pictures plus sounds) of Disgust, Erotica, Fear, Happiness, and Sadness.

**Results:**

The scale scores were elevated in both patient groups, but were not correlated with ES2 parameters. Compared to healthy controls, BD I showed prolonged ES2 latency under Erotica, and their perceived happiness and sadness intensities were negatively correlated with the respective ES2 durations, while BD II showed prolonged ES2 latencies under Disgust and Happiness, and shortened ES2 durations under Disgust, Happiness and Sadness. Moreover, ES2 duration under Sadness was significantly shorter in BD II than that in BD I.

**Conclusions:**

The cortico-brainstem inhibitory dysfunctions in BD I and BD II was different, and this difference was independent of the patient’s ongoing emotions. Our study thus provides some hints to distinguish the two types of bipolar disorders.

## Background

Bipolar disorder is generally characterized by alternating mood episodes of opposite polarity, specifically the emotional highs (mania or hypomania) or lows (depression) or the mixed states. These clinical symptoms vary between the two major types of this disease, namely, bipolar disorder type I (BD I) and type II (BD II), as well as between individuals [1]. In depressive or mixed episodes, and conditions associated with aggression/ impulsivity, the major risk for the patients is suicidal ideations and attempts [2, 3]. During manic/ hypomanic episodes, patients are usually unable to correctly judge the negative consequences of the excessive involvement in potentially harmful activities in their lives [4]. Additionally, due to symptom overlap, the two types of bipolar disorder are difficult to separate accurately. Compared to BD I, BD II is characterized by increased frequency of depressive episodes, equal or increased rates of disability, and increased risks of suicidal behavior [5]. Unfortunately, the medications available to treat these disorders are not optimally effective and their effects vary between BD I and BD II [6]. All these studies imply that there are affective, cognitive and behavioral differences between the two bipolar disorder types, which might influence their psychological and pharmacological management in the clinic. Therefore, there is a practical need to delineate the two bipolar types, for instance, by typifying them with more neurophysiological endophenotypic markers.

Researchers worldwide have identified some potential differences between BD I and BD II including genetic [7–9], anatomical [10–12], and clinical [13, 14] aspects. For instance, clinically, BD I patients presented increased sexual thoughts, desire, and activity [15, 16], while BD II patients often displayed diminished libido and sexual dysfunction [17]. In neuroimaging studies, during a negative-emotional task, BD I patients displayed a reduced activation of the left insula coupled with a reduced activation of the right supramarginal gyrus [18]. The diffusion tensor imaging techniques have also shown that the functional connectivity between structures in the fronto-limbic networks was reduced in bipolar disorders, especially in BD I, and the reduction was independent of the serotonin transporter function [19]. The neurophysiological evidence has also demonstrated higher ratios of P50 cerebral potential in BD I patients with and without a history of psychosis than those in BD II patients [20].

When processing emotions in bipolar disorder, cerebral dysfunctions were found in interactions between the frontal and temporal lobes, insula and subcortical structures such as amygdala, basal ganglia, hypothalamus and brainstem [21]. For instance, the abnormal structure and function of cortical and subcortical networks were directly related to the emotional suppression and reappraisal in bipolar disorder [22]. When facing emotional stimuli of fear and happiness, BD I patients showed increased activation in frontal regions, including the anterior cingulate cortex, dorsolateral prefrontal cortex, and amygdala [23]. In addition, when processing external negative emotions, BD I manic patients displayed significantly lower amplitude of the cerebral P3 potential compared to healthy volunteers [24]. However, there was less evidence regarding the cerebral activation when responding to facial or external emotional-stimuli in BD II patients.

On the other hand, the emotional processing involves multi-level interactions between the brainstem and cortex, and emotional expressions of complex, organized somatic and visceromotor activities are generated from brainstem structures [21]. One question arises – how about the brainstem or brainstem-cortical activities when responding to external emotions in bipolar disorder? A way of illustrating these functions might be through the assessment of brainstem reflexes under cortical drive. In normal men, there is a neurophysiological test which reflects the function of the brainstem inhibitory interneurons, that is, when applying slight painful electrical stimuli to the labial commissure, two successive exteroceptive suppressions (ES1 and ES2) of the jaw-closing muscles (masseter and temporalis) are elicited. ES1 is believed to be mediated by an oligosynaptic circuit in the pontine area, and ES2 by a polysynaptic circuit probably located in the lateral tegmental field [25]. The ES2 duration was decreased in patients with chronic tension-type headache [26], dystonia [27], stroke [28], generalized anxiety disorder [29], Alzheimer’s disease [30], Parkinson’s disease [31], and in participants with high aggression trait [32]. Notably, ES2 duration was prolonged and its latency was shortened under relaxation or depressive mood [33]. The abnormal temporalis ES2 in these neuropsychiatric disorders also supports the notion that the brainstem inhibitory reflex is influenced by the emotional-processing centers in the limbic system, such as amygdala, periaqueductal gray matter, and hypothalamus [33].

Accordingly, studying the exteroceptive suppression activities in response to external stimuli of positive or negative emotions in different types of bipolar disorder may help to better understand and diagnose the symptoms of this disease. Based on the above-mentioned research, in the current study, we have hypothesized that the external emotional-stimuli reduce the temporalis ES2 duration in both BD I and BD II patients. Therefore, we have asked both BD I and BD II patients and healthy volunteers to undergo the temporalis exteroceptive suppression tests under external stimuli of both positive (e.g., happiness and erotica) and negative (disgust, sadness and fear) emotions, to test whether ES2 durations are different in BD I and BD II under different emotional-stimuli. Additionally, the participants also answered the Mood Disorder Questionnaire [34], the Hypomania Checklist-32 [35] and the Plutchik-van Praag Depression Inventory [36] to characterize their own ongoing affective states.

## Methods

### Participants

Ninety participants were enrolled in this study: 40 healthy volunteers (24 females, 16 males; mean age: 20.33 ± 2.18 years, range: 17–28 years) were recruited from the university, hospital staff or community; 30 patients with BD I (19 females, 11 males; mean age: 20.27 ± 2.00 years, range: 18–27 years), and 20 BD II (14 females, 6 males; mean age: 20.30 ± 2.25 years, range: 18–26 years) were recruited from a psychiatric clinic. Patients were diagnosed according to the Diagnostic and Statistical Manual of Mental Disorders, Fifth edition (the DSM-5) criteria [1] by an experienced psychiatrist (WW), and they had to be medication-free for at least a month. All participants were confirmed to have no other confounding factors including schizophrenia, schizoaffective disorder, nor prior history of head injury, alcohol or tobacco abuse, psychoactive substance abuse, central nervous system inflammation, nor other neurocognitive disorders through a semi-structured clinical interview. Two master candidates (Qisha Zhu & Jiawei Wang) were available to aid participants (including the hyperactive BD I patients) in the proper filling of the required demographic information, questionnaires and the informed consents, and to ensure corrective feedbacks. The study protocol was approved by a local Ethics Committee and all participants had given their written informed consents (the informed consents of the young adolescents were signed by their guardians).

### Questionnaires

All subjects were asked to complete the following three questionnaires in Chinese in a quiet room:

A. The Mood Disorder Questionnaire [34]. It consists of three parts, including 13 forced-choice (yes or no) questions to evaluate the presence of symptoms and behaviors related to mania or hypomania, one question to determine whether two or more symptoms have been experienced at the same time, and one question to determine the extent to which symptoms have caused functional impairment on a scale ranging from “no problems” to “serious problems”. Its internal reliability was .79 in a Chinese population sample [37].
B. The Hypomania Checklist-32 [35]. It is a self-assessment tool comprising 32 items for detecting hypomanic symptoms. Individuals were instructed to answer the forced-choice (yes or no) questions about emotions, thoughts, or behaviors, and to answer questions regarding the duration, the impact on family, social and work life, or people’s reactions. Its internal reliability was .88 in a Chinese population sample [38].
C. The Plutchik-van Praag Depression Inventory [36]. It contains 34 items; each item has three scale points (0, 1 and 2), which correspond to increasing depressive tendencies. Subjects have ‘possible depression’ if they score between 20 and 25, and ‘depression’ if they score more than 25. Its internal reliability was .93 in a Chinese population sample [39].

### External emotional-stimuli

The external emotional-stimuli (i.e., the composed situational-episodes of emotion) were delivered synchronically with the temporalis exteroceptive suppression tests conducted on each participant (see below). These external emotional-stimuli were composed of pictures and sounds of the same domain saturated with high arousal and levels of emotional valence. Pictures were from the International Affective Picture System [40] and sounds were from the International Affective Digitized Sounds [41]. The five external emotional-stimuli were Disgust (picture code: 9325; sound code: 255), Fear (3053; 275), Erotica (4680; 205), Happiness (2040; 110), and Sadness (2205; 295). Additionally, two empty (silent) counterparts of these external emotional-stimuli, which were devoid of any picture or sound, called Blank 1 and Blank 2, were included. Blank 1 was presented first, and the rest episodes of external emotional-stimuli (including Blank 2) were randomly presented one-by-one afterwards. In each domain of external emotional-stimulus, a picture was presented on a screen 1.8 m in front of participants (1.4 m × 1.0 m, visual angle: 10° × 16°; lasted for 100 s); at the same time, a sound (6 s in duration, 40–50 dB in intensity, repeatedly delivered and lasted for 100 s) was delivered via headphones. After exposure to each external emotional-stimulus, participants were asked to report their perceived intensity to that emotion, with a visual analogue scale (VAS) which was ranged from 0 (none) to 10 (most intense).

### Neurophysiologic assessment

During the procedure, participants sit in a reclining armchair. Exteroceptive suppressions of temporalis muscle activity were recorded under each external emotional-stimulus (or Blank 1 or Blank 2), according to previous methods [25]. Briefly, surface recording electrodes (NE-132B, 0.9 cm in diameter) were used, the active one was placed over the anterior belly of the left temporalis muscle approximately 0–0.5 cm in front of the temporal hairline, and the reference one was placed just in front of the tragus and the ground electrode was placed on the left arm. The electrical impedances of each electrode were kept ≤10 kΩ. Electrical stimuli of 20 mA intensity and 0.2 ms duration were applied over the ipsilateral labial commissure during maximal voluntary tooth-clenching, with an inter-stimulus interval of about 10 s, and the electrical stimuli were applied ten times under each external emotional-stimulus.

The electromyography (EMG) was recorded with a Nihon Kohden-Neuropack sigma device (band pass: 20 Hz-2 kHz). The baud rate in the device was set to 9600 bit/s. Ten rectified responses were averaged over a 200-ms time period. In keeping with a suggestion by Schoenen [25], we chose the 80% magnitude of suppression (or 20% prestimulus EMG level) criterion to determine ES1 and ES2 onsets and offsets in each individual. ES1 was measured between 5 and 30 ms, and ES2 between 40 and 100 ms after stimulation. Because both the onsets and offsets of very short exteroceptive suppression periods were difficult to determine, duration ≤5 ms was unclear and thus considered as 0 ms. Data collected under Blank 1 sessions served as baseline control (before external emotional-stimuli), while those collected under Blank 2 sessions served as another control to washout the possible effects of the external emotional-stimuli.

### Data analyses and statistics

Mean ES1 and ES2 latencies and durations under different external emotional-stimuli (and Blanks 1 and 2) in the three groups were subjected to two-way ANOVA. In addition, the mean VAS scores of perceived intensity during external emotional-stimuli in the three groups were also subjected to two-way ANOVA, while the mean scale scores of MDQ, HCL-32 and PVP in the three groups were subjected to one-way ANOVA. Once a significant major (group) effect was found, post-hoc analysis by the Fisher Least Significant Difference test and the Bonferroni correction were employed to evaluate between-group differences. A *p* value less than .05 was considered as significant. With the present sample size, the power to detect an effect was larger than 80% at this *p* level. The Spearman rank order correlation was used to search for the possible relationships between the ES1/ ES2 latency and duration, VAS, MDQ, HCL-32, and PVP scores. In order to reduce the risk of Type I error, *p* < .01 was considered as significant regarding correlations.

## Results

### Demographic and clinical aspects

There was no significant difference regarding either age (one-way ANOVA, *F* [2, 87] = .01, *p* = .99, MSE = .029), education level (*F* [2, 87] = .33, *p* = .72, MSE = .34), or gender (Pearson *χ* ^*2*^ = .57, *p* = .75) distribution between groups. The mean PVP scores were significantly different among the three groups (*F* [2, 87] = 53.47, *p* < .001, mean square effect (MSE) = 1881.66), with that of the BD II group being higher than those of the BD I (*p* < .05, 95% confidence interval (CI) = 12.90 ~ 19.35) and control (*p* < .05, 95% CI = 6.14 ~ 9.11) groups. The mean MDQ scores were also significantly different among the three groups (*F* [2, 87] = 49.24, *p* < .001, MSE = 280.07), with that of the BD I group being higher than those of the BD II (*p* < .05, 95% CI = 3.66 ~ 6.40) and controls (*p* < .05, 95% CI = 4.26 ~ 6.55) groups. The mean HCL-32 scores were also significantly different among the three groups (*F* [2, 87] =57.29, *p* < .001, MSE = 549.21), with that of the BD I group being higher than those of the BD II (*p* < .05, 95% CI = .02 ~ 3.58) and control (*p* < .05, 95% CI = 6.14 ~ 9.11) groups (Table 1).

**Table 1:** Scale scores (mean ± S.D.) of the Plutchik-van Praag Depression Inventory, the Mood Disorder Questionnaire, and the Hypomanic Checklist-32 in healthy volunteers (controls, *n* = 40), bipolar I (BD I, *n* = 30) and II (BD II, *n* = 20) disorder patients

	Controls	BD I	BD II
Plutchik-van Praag Depression Inventory	8.88 ± 6.00	10.40 ± 5.93	25.00 ± 5.80^a,b^
Mood Disorder Questionnaire	4.33 ± 3.08	9.73 ± 1.23^a^	4.70 ± 2.05^b^
Hypomanic Checklist-32	14.88 ± 4.15	22.5 ± 1.33^a^	20.70 ± 2.43^a,b^

^a^ *p* < .05 vs. Healthy controls; ^b^ *p* < .05 vs. BD I

### Neurophysiological test under external emotional-stimuli

**Fig. 1:**
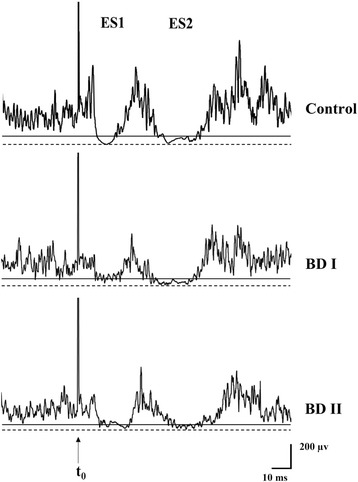
Illustrative recordings of temporalis exteroceptive suppressions in a healthy volunteer (Control) under Blank 1, a patient with bipolar disorder I (BD I) under Erotica, and a patient with bipolar disorder II (BD II) under Sadness. Dotted line stands for the complete (100%) inhibition, and solid line the partial (80%) inhibition of the respective prestimulus EMG activity

Illustrative recordings of the temporalis exteroceptive suppression in the three groups are shown in Fig. 1. There were significant differences in ES2 durations among the three groups (group effect, *F* [2, 87] = 3.18, *p* = .047, MSE = 1519.22) (Table 2). In the BD I group, ES1 latencies under Erotica (*p* < .05, 95% CI = .17 ~ 2.25), Fear (*p* < .05, 95% CI = .40 ~ 2.79), Happiness (*p* < .05, 95% CI = .21 ~ 2.08) and Blank 2 (*p* < .05, 95% CI = .17 ~ 2.84) were significantly more prolonged than those in the control group. ES2 latencies in the BD I group under Erotica (*p* < .05, 95% CI = .26 ~ 5.63) and Blank 2 (*p* < .05, 95% CI = .77 ~ 5.66) were also significantly more prolonged than those in the control group. In the BD II group, ES1 durations under Blank 1 (*p* < .05, 95% CI = .14 ~ 7.31) were significantly more prolonged than those in the BD I group. ES2 latencies under Disgust (*p* < .05, 95% CI = .27 ~ 6.11) and Happiness (*p* < .05, 95% CI = 1.15 ~ 7.52) were significantly more prolonged than those in the control group. ES2 durations under Disgust (*p* < .05, 95% CI = −10.43 ~ −.03), Happiness (*p* < .05, 95% CI = −12.23 ~ −2.57) and Sadness (*p* < .05, 95% CI = −12.68 ~ −2.79) were significantly shorter than those in the control group. Additionally, ES2 durations under Sadness (*p* < .05, 95% CI = −12.16 ~ −1.73) in the BD II group was significantly shorter than those in the BD I group.

**Table 2:** Latencies and durations (in ms, mean ± S.D.) of temporalis muscle exteroceptive suppressions under external emotional-stimuli in healthy volunteers (controls, *n* = 40), bipolar I (BD I, *n* = 30) and II (BD II, *n* = 20) disorder patients

	ES1		ES2	
	Latency	Duration	Latency	Duration
Controls				
Blank 1	12.94 ± 1.67	11.54 ± 4.62	49.03 ± 5.64	31.49 ± 9.34
Disgust	13.00 ± 1.38	11.01 ± 5.06	45.49 ± 6.07	32.46 ± 11.04
Erotica	13.02 ± 1.79	11.64 ± 4.86	46.62 ± 5.45	31.17 ± 11.57
Fear	12.47 ± 1.43	11.59 ± 5.00	47.37 ± 5.73	30.16 ± 11.52
Happiness	12.73 ± 1.49	11.05 ± 5.41	47.08 ± 5.28	32.29 ± 10.37
Sadness	13.27 ± 2.23	11.45 ± 5.15	46.26 ± 5.74	31.03 ± 9.57
Blank 2	13.05 ± 1.71	9.95 ± 5.44	46.14 ± 5.25	31.62 ± 10.09
BD I				
Blank 1	13.72 ± 2.66	9.90 ± 4.62	50.29 ± 6.13	28.84 ± 9.15
Disgust	13.62 ± 2.91	11.16 ± 4.87	47.29 ± 4.96	31.91 ± 8.77
Erotica	14.22 ± 2.98^a^	11.19 ± 4.47	49.57 ± 4.96^a^	28.44 ± 9.01
Fear	14.07 ± 3.16^a^	9.87 ± 4.80	49.55 ± 4.84	27.80 ± 10.23
Happiness	13.87 ± 2.77^a^	10.58 ± 5.10	49.15 ± 4.96	29.60 ± 7.50
Sadness	13.83 ± 2.81	10.75 ± 4.47	48.01 ± 4.80	30.25 ± 8.08
Blank 2	14.55 ± 4.31^a^	10.41 ± 4.72	49.35 ± 4.84^a^	28.70 ± 8.24
BD II				
Blank 1	14.13 ± 4.35	13.63 ± 10.12^b^	50.83 ± 8.79	27.04 ± 10.74
Disgust	13.01 ± 1.28	11.51 ± 4.64	48.68 ± 4.28^a^	27.24 ± 7.08^a^
Erotica	13.20 ± 1.15	10.00 ± 4.72	49.25 ± 6.65	25.98 ± 9.23
Fear	13.41 ± 3.01	10.27 ± 5.17	49.92 ± 5.58	25.13 ± 8.21
Happiness	12.95 ± 0.99	10.38 ± 4.82	51.42 ± 7.90^a^	24.89 ± 7.29^a^
Sadness	12.80 ± 1.13	11.64 ± 4.47	48.84 ± 4.81	23.30 ± 9.50^a,b^
Blank 2	13.18 ± 1.01	11.34 ± 4.75	48.24 ± 5.12	26.74 ± 7.94

^a^ *p* < .05 vs. Healthy controls; ^b^ *p* < .05 vs. BD I

Regarding the perceived intensities of the external emotional-stimuli, the sample size of 90 participants in the current study resulted in an internal alpha of .79 for the VAS measurement. The VAS scores were also statistically significantly-different among the three groups (group effect, *F* [2, 87] = 4.46, *p* = .014, MSE = 47.40). The VAS scores under Disgust (*p* < .05, 95% CI = .37 ~ 2.36) and Fear (*p* < .05, 95% CI = .40 ~ 2.58) were significantly higher in the BD I group than those in the control group. In the BD II group, VAS scores under Disgust (*p* < .05, 95% CI = .35 ~ 2.60) were significantly higher than those in the control group, VAS scores under Sadness (*p* < .05, 95% CI = −2.37 ~ −.22) were significantly lower than those in the BD I group (Table 3).

**Table 3:** Visual analogue scale scores (mean ± S.D.) of perceived emotional intensity under external stimuli in healthy volunteers (controls, *n* = 40), bipolar I (BD I, *n* = 30) and II (BD II, *n* = 20) disorder patients

	Disgust	Erotica	Fear	Happiness	Sadness
Controls	5.20 ± 2.14	5.55 ± 2.07	5.86 ± 2.46	5.06 ± 2.14	4.99 ± 1.82
BD I	6.57 ± 1.87^a^	6.27 ± 1.54	7.35 ± 1.95^a^	6.00 ± 1.55	5.77 ± 1.63
BD II	6.68 ± 2.20^a,b^	6.10 ± 1.62	6.90 ± 2.31	5.25 ± 2.15	4.48 ± 2.28^b^

^a^ *p* < .05 vs. Healthy controls; ^b^ *p* < .05 vs. BD I

### Relationship test between variables

Significant correlation was found between the ES2 duration and VAS score under Happiness (*r* = −.53, *p* < .01) and Sadness (*r* = −.49, *p* < .01) in the BD I group (*n* = 30). However, no other significant relationship between ES1 and ES2 latencies/ durations and PVP, MDQ or HCL-32 scale scores was found in a given group.

## Discussion

In the current study, after controlling for age, gender and education level factors, we found that patients with BD II scored significantly higher than the healthy volunteers and patients with BD I did on PVP; patients with BD I scored significantly higher than the healthy volunteers and patients with BD II did on MDQ. We also found that patients with BD I and BD II scored significantly higher than the healthy volunteers, while patients with BD I scored higher on HCL-32 than those with BD II. These outcomes are consistent with previous findings [42–44] and support the suggestion that BD I and BD II represent manic and depressive extremes in the bipolar spectrum respectively [45]. Moreover, we found that ES2 latency was reduced under Erotica in the BD I group, ES2 latencies were prolonged under Disgust and Happiness, and ES2 durations were reduced under Disgust, Happiness and Sadness in the BD II group. Furthermore, there was no relationship between ES1 and ES2 latency and duration and PVP, MDQ or HCL-32 scores in a given group, suggesting that the abnormal brainstem inhibitory reflexes were independent from their clinical, ongoing affective states. To the best of our knowledge, this is the first time that the temporalis ES2 methodology is tried under different stimuli of external emotions to examine the brainstem dysfunctions in the two types of bipolar disorder.

Regarding BD I, patients often display cognitive impairment not only during their own different mood episodes but also during their euthymic phases [46, 47]. Patients often present their own affective states as mania, depression or mixture of both [18, 48], they also consistently show increased sexual thoughts, desire, and activity [15, 16]. The perception, cognition and behavior under erotic stimulation, might be linked to more excitation of cerebral areas such as the prefrontal, hypothalamus and cingulate cortices [49], which in turn delayed the activation of brainstem inhibitory interneurons, thus delaying the latencies of ES1 and ES2 under erotica. On the other hand, when processing both fear and happiness in patients with BD I, increased activation of the left amygdala/ ventrolateral prefrontal cortex [50], thalamic, pallidal, and caudate/ putamen regions [51] were reported, which again might be the reason for the prolonged ES1 latencies under fear and happiness. Indeed, our BD I patients perceived higher intensity under Fear. Apparently, the delayed activation had kept its effect under Blank 2, where both ES1 and ES2 latencies were prolonged. Moreover, BD I participants displayed normal ES1 and ES2 durations, but their perceived happiness and sadness intensities were negatively correlated with the ES2 duration. Possible reasons might be that the cerebral areas involved when processing both positive (such as happiness) and negative (such as sadness) emotions had functioned excessively to inhibit the brainstem inhibitory interneurons. Previous studies did show that bipolar disorders (mainly BD I) often presented elevated emotions such as happiness, disgust and sadness [52].

Compared to BD II, the ES1 duration under Blank 1 was significantly decreased in BD I patients, which implied that the inhibitory oligosynaptic interneurons were less activated at the baseline (resting) state in the disorder. Although the inhibitory polysynaptic interneurons mediating ES2 duration were less affected in BD I, this finding was in accordance with the baseline manic state in this pathology [4].

Regarding BD II, the ES2 durations were reduced under Disgust, Happiness and Sadness in the current study. Indeed, fMRI studies have demonstrated the involvement of several cerebral areas when these emotions were processed in BD II. For instance, when responding to facial disgust, there was altered connection between prefrontal-subcortical network, diminished prefrontal and increased limbic areas in both BD I and BD II patients [53]. When processing happy faces in BD II patients, there was a loose connection between the orbitomedial prefrontal cortex and amygdala, which might result in an abnormal activation of the latter nucleus [54]. When processing sadness, BD II patients showed a reduced regional orbitofrontal and limbic activations and the magnitude of these activations were correlated with depression severity [55], and showed an increased amygdala activation in another study [56]. Interestingly, in our study, BD II patients perceived higher intensity under Disgust. These results pointed to the excessive activation of brain nuclei which might cause more inhibition to the brainstem interneurons mediating ES2 under these emotions.

Moreover, in BD II patients, ES2 latencies were prolonged under Disgust and Happiness. Similarly, the above-mentioned subcortical nuclei under Disgust and Happiness might inhibit the activation onset of the brainstem inhibitory interneurons, thus they might result in delayed ES2 in BD II patients. However, the possibly abnormal activation of the left hippocampal area failed to exert its control effect on the activation-onset of brainstem inhibitory interneurons, thus allowing an intact ES2 latency under Sadness in our BD II group. Although it is still unknown how much the cognition contributes to the ES2 formation, the memory processing function of the hippocampus [57] might help to facilitate the ES2 activation in this disorder. Indeed, clinically, BD II patients more depression tendency [1] and might be more adapted to the depressive situation [45, 58]. Additional support for the reduced ES2 under Sadness in the BD II group might come from the findings that chronic tension-type headache had reduced ES2 duration [26, 59], and this headache type was often comorbid with depression [60].

However, it must be noted that our study suffers from at least four design flaws. First, we only enrolled bipolar disorder patients; recruiting other non-bipolar disease-controls, such as unipolar major depression and schizophrenia, which also display depressive or manic episode, would be nice justifications for our findings. Second, we did not record the personality traits or personality disorder functioning styles of our participants; whether their personalities contribute to our current findings remains unknown. Third, we only employed five emotions as external stimuli; additional one, such as surprise, anger, or contempt might also display their effects on the brainstem reflexes. One more concern is that ES1 latencies and durations showed some alterations in both the BD I and BD II groups. Since ES2, rather than ES1, reflects more about the brainstem functions, and provides more meaningful information in this regard [25], the outcomes in the current study nevertheless provide some hints about the cortico-brainstem dysfunctions under different external emotional-stimuli in different types of bipolar disorder. Regarding the temporalis ES2 reduction, Erotica exerted a pronounced effect on BD I patients, while Disgust, Happiness and Sadness exerted pronounced effects on BD II patients. From a neuropsychophysiological aspect, on the one hand, our results help to delineate the two types of bipolar disorder, and on the other, they offer different therapeutic modalities of emotional control for them. In spite of these clinical implications, our current findings would need further confirmation from other independent laboratories worldwide.

## Abbreviations

BD I: Bipolar I
BD II: Bipolar II
EMG: Electromyography
ES: Temporalis exteroceptive suppression
HCL-32: The Hypomania Checklist-32
MDQ: Mood Disorder Questionnaire
PVP: The Plutchik-van Praag Depression inventory
VAS: Visual analogue scale
